# Effects of carbon concentration, oxygen, and controlled pH on the engineering strain *Lactiplantibacillus casei* E1 in the production of bioethanol from sugarcane molasses

**DOI:** 10.1186/s13568-021-01257-x

**Published:** 2021-06-27

**Authors:** Song Wang, Ran Tian, Buwei Liu, Hongcai Wang, Jun Liu, Chenghui Li, Mingyue Li, Smith Etareri Evivie, Bailiang Li

**Affiliations:** 1 Food College, Northeast Agricultural University, Harbin, 150030 China; 2 Shandong Yuwang Ecological Food Industry Co., Ltd, Dezhou, 251200 Shandong China; 3 Department of Animal Science, Faculty of Agriculture, University of Benin, Benin City, 300001 Nigeria; 4 Department of Food Science and Human Nutrition, Faculty of Agriculture, University of Benin, Benin City, 300001 Nigeria

**Keywords:** Sugarcane molasses, *Lactiplantibacillus casei*, Bioethanol, Fermentation

## Abstract

Sugarcane molasses are considered a potential source for bioethanol’s commercial production because of its availability and low market price. It contains high concentrations of fermentable sugars that can be directly metabolized by microbial fermentation. Heterofermentative lactic acid bacteria, especially *Lactiplantibacillus casei*, have a high potential to be a biocatalyst in ethanol production that they are characterized by strong abilities of carbohydrate metabolism, ethanol synthesis, and high alcohol tolerance. This study aimed to evaluate the feasibility of producing ethanol by *Lactiplantibacillus casei* used the ethanologen engineering strain *L. casei* E1 as a starter culture and cane molasses as substrate medium. The effects of environmental factors on the metabolism of *L. casei* E1 were analyzed by high-performance liquid chromatography (HPLC) system, and the gene expression of key enzymes in carbon source metabolism was detected using quantitative real-time PCR (RT–qPCR). Results showed that the strain could grow well, ferment sugar quickly in cane molasses. By fermenting this bacterium anaerobically at 37 °C for 36 h incubation in 5 °BX molasses when the fermenter’s pH was controlled at 6.0, ethanol yield reached 13.77 g/L, and carbohydrate utilization percentage was 78.60%. RT-qPCR results verified the strain preferentially ferment glucose and fructose of molasses to ethanol at the molecular level. In addition, the metabolism of sugars, especially fructose, would be inhibited by elevating acidity. Our findings support the theoretical basis for exploring Lactic acid bacteria as a starter culture for converting sugarcane molasses into ethanol.

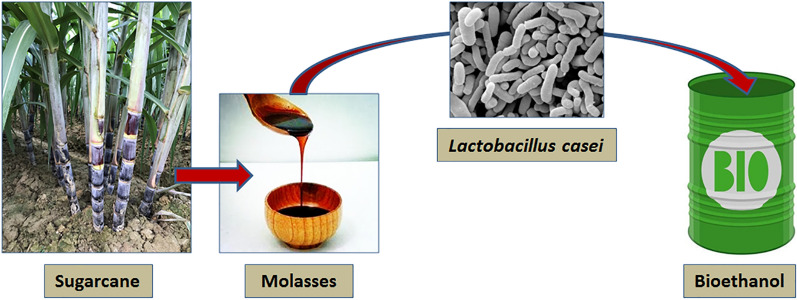

## Key points

1. Verificating the ability of engineering strain *L. casei* E1 to produce bioethanol.
2. The possibility of lactic acid bacteria as a starter culture for ethanol production.
3. Carbohydrate metabolic characteristics of the strain in sugarcane molasses.

## Introduction

Bioethanol, which can serve as a gasoline additive to increase octane and improve vehicle emissions in its pure form (Baki et al. 2020), is an alcohol made by microorganisms through the fermentation of carbohydrates. Bioethanol can be derived from cellulosic biomass such as trees and grasses, which are non-food sources. However, since the high cost of hydrolyzing lignocellulose biomass and lower ethanol titers and yields, the development of bioethanol production have been limited (Fan et al. 2018). Additionally, the cellulose and hemicellulose of fibrous biomass are difficult to convert into fermentable sugars for direct use by microorganisms to produce ethanol. Also, phenols, aldehydes, acids, and other organic substances produced during pretreatment and hydrolysis may inhibit fermentation and have toxic effects on the biocatalytic host (Tanawut et al. 2020). This results in low efficiency and the need to integrate additional removal and recovery processes during fermentation.

Sugarcane molasses is a subsidiary product of the sugar industry, containing approximately 50% of fermentable sugars (sucrose, glucose, and fructose), protein, vitamins, and trace elements that can be directly metabolized by microbial fermentation (Lino et al. 2018). Thus, it doesn’t require additional operations that significantly increase the cost of biosyntheses such as chemical hydrolysis or enzymatic hydrolysis (Dziugan et al. 2013), and has been extensively exploited as alternative lowcost feedstock. It can be used as substrate for the production of lactic acid, succinic acid, citric acid, butanol, etc. Currently, sugarcane molasses main use is in the production of fuel ethanol (Lino et al. 2018).

*Saccharomyces* has been the biocatalyst of preference for ethanol production. However, it typically lacks the ability to utilize pentose sugars (Jarboe et al. 2007), which is important in lignocellulosic-based alcohol production. Additionally, *S. cerevisiae* only secretes enzymes at low levels (Wang et al. 2013) and is sensitive to compounds generated during biomass pretreatment and hydrolysis (Parreiras et al. 2014). As has many advantages (higher sugar absorption rate, wider variety of available carbohydrates, easier to genetic engineering or higher tolerance, etc.), bacteria such as *Zymomonas mobilis* and *Escherichia coli* have been extensively studied as potential biocatalysts for alcohol production (Jarboe et al. 2007).

*Lactiplantibacillus casei*, one species of *Lactiplantibacillus* that has been widely used in the food industry over decades, grows well in carbohydrate-rich environments and converts diverse substrates to organic acids and ethanol since it can perform heterolactic fermentation (Zotta et al. 2018). *L. casei* is characterized by strong abilities of carbohydrate metabolism (Koryszewska-Bagińska et al. 2019) and a wide range of substrate utilization such as monosaccharides, disaccharides, various oligosaccharides, and polysaccharides (Cai et al. 2007; Suzuki et al. 2020). *L. casei* can metabolize and synthesize ethanol through the Embden Meyerhof Parnas (EMP) pathway, and some strains can also produce ethanol through heterogeneous fermentation Entner-Doudoroff (ED) pathways. More importantly, *L. casei* boasts of several relatively simple metabolism pathways (Xin et al. 2018) and genetic advantages, including the availability of genome sequences, genome-scale metabolic models and methods for integration of foreign DNA (Welker et al. 2015; Blanco-Míguez et al. 2019; McAuliffe et al. 2019). Recently, it has been found that *L. casei* is able to grow on food-wastes. Ricciardi et al (2019) incubated *L. casei* N87 in cheese whey permeate which is a low-cost feedstock used for the production of biomass, the results showed that the strain grew well and produced lactate, acetate, etc. These attributes suggest that *Lactiplantibacillus casei* has a high potential to be a biocatalyst in bioethanol production.

The growth and metabolism of strains are affected by many factors such as carbon, nitrogen, temperature, pH, and oxygen (Zotta et al. 2017; Meng et al. 2019). The dilution of the natural substrate is directly related to the concentration of each component of the medium. Excessive concentration of the natural substrate leads to high concentrations of carbohydrates, which will increase osmotic pressure that changes or destroys the abilities of strains to transport and metabolic each component (Bubnová et al. 2014). In environments with different oxygen content, since the metabolic pathways of strains change, aerobic and anaerobic fermentation will affect types and yields of products (Dittrich et al. 2005; Wushke et al. 2017; Matsuoka and Kurata 2017). The effects of oxygen on products of *L. casei* strain using food-wastes substrate had be genetically verified and analyzed in detail (Ricciardi et al. 2019). *L. casei* synthesizes various organic acids such as pyruvate, lactic acid, acetic acid, and succinic acid (Vinay-Lara et al. 2016). Fermentation progresses, organic acids that can increase the acidity of fermentation liquid gradually accumulate. The dramatic change in pH will affect the carbohydrate metabolism of strains because high acidity will cause metabolic-related components on the cell membrane, such as channel proteins, transport proteins, and signal pathway proteins, to lose their normal function (Virgilio et al. 2017).

The metabolism of carbohydrates by the strain mainly depends on the catalysis of metabolic-related enzymes. Comparative genomic analysis results revealed that the wild-type strain *L. casei* 12A could degrade nine sugars in cells (Wang et al. 2015). The strain first used glucokinase (GK, EC 2.7.1.2) to degrade glucose to 6-phosphate glucose, which is the common intermediate product and intersection of various metabolic pathways, including glycolysis pathway (EMP), pentose phosphate pathway (PPP), glycogen synthesis pathway, and decomposition pathway. Therefore, GK is one of the key enzymes for glucose metabolism. Second, the strain could use phosphofructokinase (PFK, EC 2.7.1.56) to convert fructose to 1,6-fructose diphosphate (FDP) and then facilitate its entrance into the glycolysis pathway. PFK is one of the key enzymes in fructose metabolism. Third, the strain hydrolyzed sucrose to glucose and fructose using invertase (INV, EC 3.2.1.26). INV is one of the key enzymes for the metabolism of sucrose. Therefore, the three key enzymes' expression levels were detected to investigate the effect of some factors on carbon source metabolism.

In this study, one strain *L. casei* E1, which was engineered from wild strain *L. casei* 12A to produce ethanol instead of lactate as its major end-product from carbohydrates (Vinay-Lara et al. 2016), was selected to explore the effects of environmental factors on the metabolisms. Using sugarcane molasses solution as medium, the living cell number, the carbohydrates utilization, and product synthesis abilities of the strain under different conditions were analyzed. The types and yields of carbohydrates and products in fermentation liquid were determined by the high-performance liquid chromatography (HPLC) system. Quantitative real-time PCR (RT-qPCR) was used to analyze the expression level of the key enzyme genes of carbohydrate metabolism and investigate the effect of some factors on the strain's carbohydrate metabolism.

## Materials and methods

### Preparation of sugarcane molasses solution

The fresh sugarcane molasses, whose initial liquid concentration is 80 °Bx, was diluted to 40 °Bx, 30 °Bx, 20 °Bx, 10 °Bx, 7.5 °Bx, and 5 °Bx, respectively, using deionized water. To ensure sufficient nitrogen sources in the fermentation solution, 5 g/L of yeast extract (Oxoid, LP0021), 5 g/L of peptone (Oxoid, LP0037B), and 10 g/L of beef extract (Oxoid, LP0029B) were added to various molasses concentrations according to the MRS (De Man, Rogosa and Sharpe) basic medium formula. Then the pH value was adjusted to 6.0.

### Construction of *L. casei* E1

The engineered *L. casei* E1, a derivative of wild strain *L. casei* 12A (GenBank: CP006690.1) lacking *ldh*1, was constructed using the procedure described by Broadbent *et al*. (2003). Briefly, a synthetic production of ethanol (PET) cassette encoding the *Zymomonas mobilis* pyruvate decarboxylase (PDC) and alcohol dehydrogenase (ADHII) genes under the control of the native *L. casei* 12A phosphoglycerate mutase (*pgm)* promoter (*L. casei* 12A Δ*ldh*1::P*pgm*-PET) was assembled and codon-optimized using Java Codon Adaptation Tool and inserted into the 12A *ldh*1 locus (Vinay-Lara et al. 2016).

### Analysis of metabolic characteristics

The utilization of carbohydrates and production of metabolic end-products were detected by the HPLC system, including Waters Alliance 2695 Spectrometer, Waters 2414 Refractive Index Detector (RID), and Aminex HPX-87H (300 mm × 7.8 mm ID, 9 μm, Bio-Rad Labors) chromatographic separation column under 5 mmol/L sulfuric acid solution of the mobile phase, 30 °C of column temperature, and 0.6 mL/min of flow rate (Qi et al. 2017).

### Culture of *L. casei* E1

The working culture of *L. casei* E1 was prepared from a frozen stock using two sequential transfers (0.1% inoculum) that the strain incubated in MRS medium statically at 37 °C for 24 h and cultured in 10 °Bx of sugarcane molasses solution at 37 °C for 30 h. Then the bacterial liquid was transferred into different concentrations of sugarcane molasses solutions, respectively, with the initial concentration of 10^7^ CFU/mL. The strain's living cell number was determined at 0, 8, 12, 24, 30, 36, 48, and 60 h on MRS solid medium.

### Effects of oxygen on ethanol production

The strain was cultured in two glass fermenters-bioreactors (5L, BIOTECH-5BG, Shanghai Biotech Biological Equipment Engineering Co., Ltd.) with the same molasses solution under anaerobic (20 psi, CO_2_/N_2_) and aerobic conditions (sterile air, stirring), respectively, at 37 °C for 72 h. The liquid fermentation samples were determined at 0, 4, 8, 12, 16, 24, 30, 36, 48, and 72 h by High-Performance Liquid Chromatography (HPLC).

### Effects of controlled and uncontrolled pH cultures on growth and metabolism

One of the fermenters' pH value was controlled by the Bioreactor control system pumping in 6 mol/L of NaOH and 6 mol/L of HCl. The pH electrode recorded the acidity of the unregulated fermentation broth. The fermentation broth was collected at 0, 4, 8, 12, 16, 24, 30, 36, 48, and 60 h for further analysis.

### Effects of controlled and uncontrolled pH cultures on expression of key enzyme genes of carbon source metabolism during transformation

The absolute gene expressions of glucokinase (GK, EC 2.7.1.2), invertase (INV, EC 3.2.1.26), and phosphofructokinase (PFK, EC 2.7.1.56) were detected through the real-time fluorescence quantitative PCR (RT-PCR) during fermentation. The primers utilized in this study have been described in Table 1. Working cultures were prepared from the frozen storage of liquid nitrogen. DNA was extracted using the Ezup column, a bacterial genomic DNA extraction kit (SK8255 Sangon). Purpose genes were recovered using a columnar DNA adhesive recovery kit (SK8131 Sangon). Connected products were transformed using the one-step rapid receptor cell preparation kit (SK9307 Sangon). The plasmid DNA was extracted using the SanPrep column plasmid DNA small amount extraction kit (SK8191 Sangon), and the RT-PCR detection was operated using ABI SybrGreen PCR Master Mix (2 ×) kit.

**Table 1:** The primers of key enzyme genes

Target	Primer	Sequence
GK	Forward	5′ ATTGAGGTGTAATAGGTCGGTGG 3′
	Reverse	5′ CGATTTTATGACGATTGATGCC 3′
INV	Forward	5′ AGACGCAGACTTGTTGTTTCCC 3′
	Reverse	5′ GACGTTAGATGATGGCGATGAG 3′
PFK	Forward	5′ CATTGCCAAAGAAGCGACC 3′
	Reverse	5′ AAGACAACGATTCATCTGCCTG 3′

### Statistical analysis

All experiments were performed at least three independent times and values are expressed as mean ± standard deviation (SD). In order to compute the relative fold changes in gene expression of the studied genes, datas were analysed using the comparative 2^−ΔΔCt^ method was used (Livak and Schmittgen 2001).

## Results

### Effects of molasses concentration on the growth of the strain

**Fig 1:**
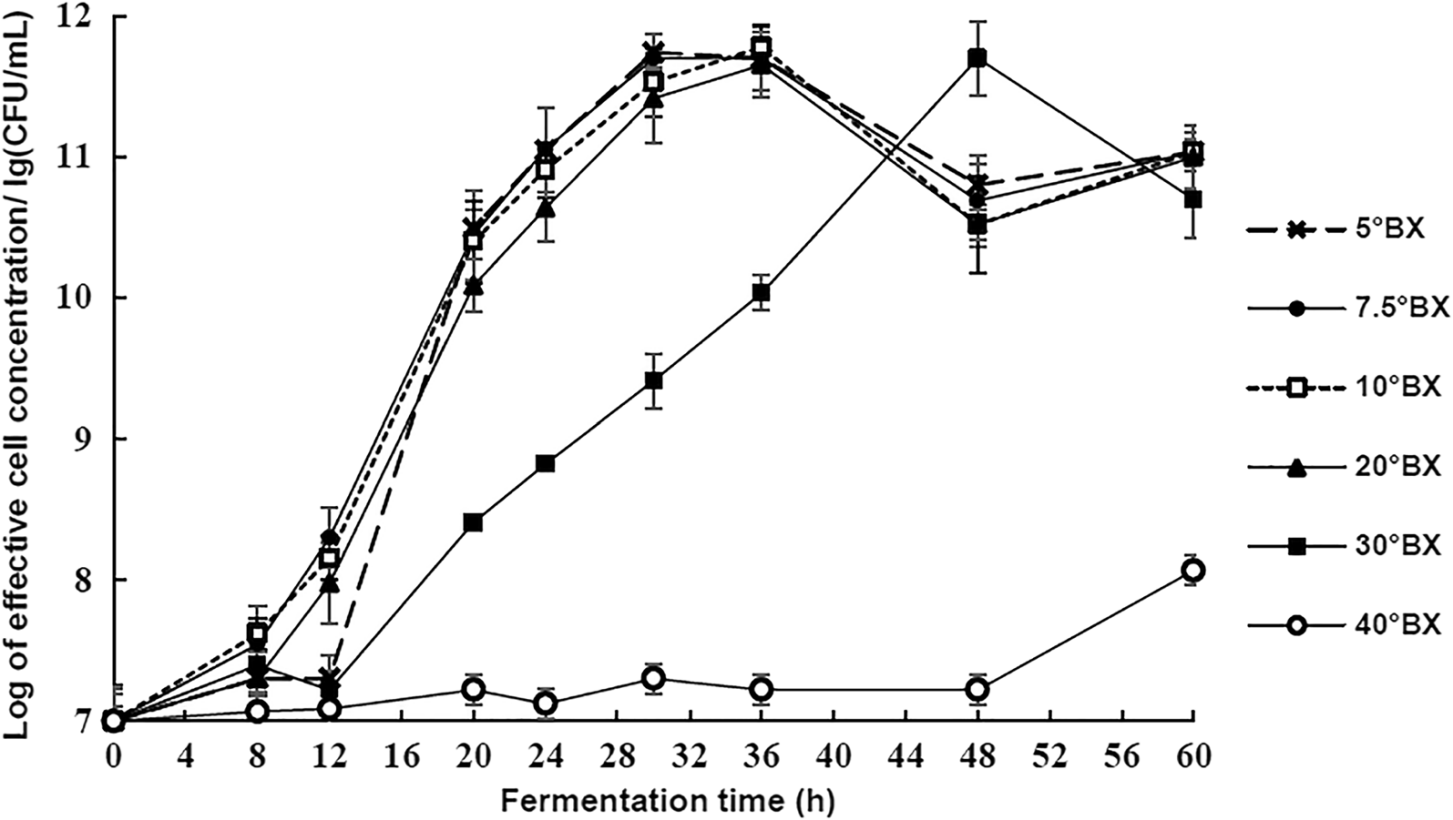
Growth of strain in different concentrations of cane molasses

As shown in Fig. 1, the growth curves of *L. casei* E1 are very similar in the four low concentrations of cane molasses (5 °Bx, 7.5 °Bx, 10 °Bx, and 20 °Bx), the colony number reached the maximum value of 10^11^ CFU/mL after about 30 h of fermentation. In addition, the growth rates of strain in logarithmic phase at low concentrations were significantly higher than those in other two high concentration mediums (30 °Bx and 40 °Bx). Although the maximum number of living cells could also reached 10^11^ CFU/mL in 30 °Bx, the strain’s slow growth resulted in needing 48 h to achieve this. Meanwhile, the strain did not grow until 48 h at 40 °Bx. The results showed that there are sufficient carbohydrates in the 5 °Bx medium for the strain fermentation. To investigate the metabolism characteristics of the strain, four low concentrations of molasses(5 °Bx, 7.5 °Bx, 10 °Bx, and 20 °Bx) were chosen.

### Effects of molasses concentration on the metabolism of strain

**Fig. 2:**
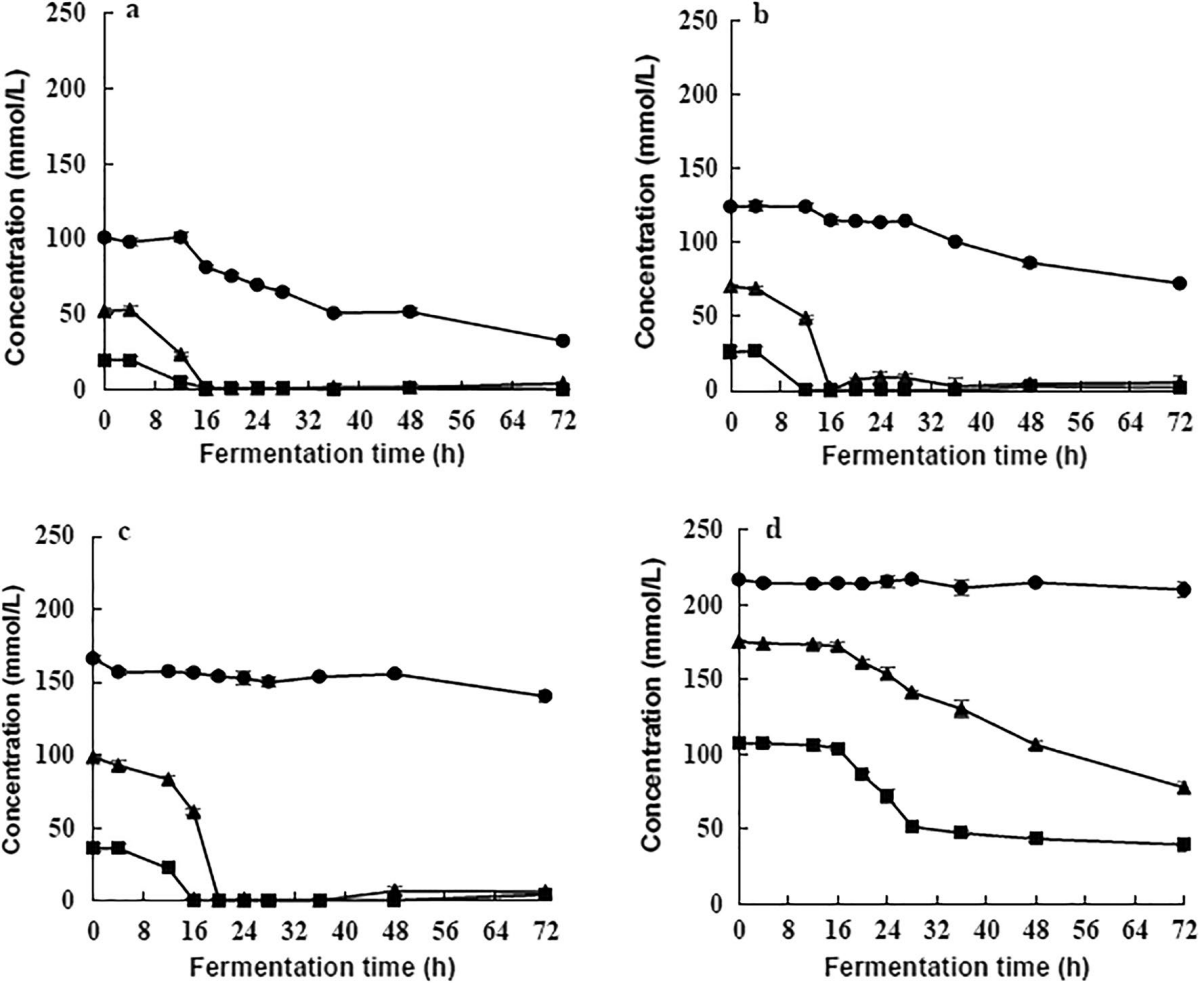
Carbohydrate utilization in different concentrations of cane molasses. Carbohydrates tested: Sucrose , Glucose , Fructose . **a** 5 °BX; **b** 7.5 °BX; **c** 10 °BX; **d** 20 °BX

It can be seen from Fig. 2 that strain E1 first metabolized glucose and fructose and then sucrose; the higher the concentration of molasses, the later the sucrose metabolism started. As shown in Fig. 2a, b, at concentrations of 5 °Bx and 7.5 °Bx, the strain consumed glucose and fructose in about 16 h, the sucrose metabolism began at 12 h. Comparing the two concentrations (5 °Bx and 7.5 °Bx), the sucrose consumption of the strain was faster at 5 °Bx, which accelerated at 16 h. While in 7.5 °Bx, sucrose consumption accelerated after 28 h (Fig. 2a, b). In 10 °Bx molasses, sucrose consumption was very low (started after 48 h). In 20 °Bx, only partial glucose and fructose were consumed (Fig. 2c, d).

The utilization rate of carbohydrates in different molasses concentrations is also used as an index to select the optimal medium concentration for bioethanol production by *L. casei* E1. The results showed that the lower the concentration of substrate, the higher the fermentation efficiency. The total sugars' utilization rate was 75.2% in 5 °Bx molasses fermented for 72 h (Table 2).

**Table 2:** Carbohydrate metabolism in four low concentrations of cane molasses

Compound (g/L)	Concentrations of fermentation broth							
	5 °BX		7.5 °BX		10 °BX		20 °BX	
	0 h	72 h	0 h	72 h	0 h	72 h	0 h	72 h
Sucrose	34.52 ± 0.26	11.03 ± 0.41	42.41 ± 1.35	24.68 ± 0.33	56.79 ± 1.04	47.92 ± 0.48	74.18 ± 0.89	71.85 ± 1.13
Glucose	3.57 ± 0.04	0.02 ± 0.01	4.67 ± 0.07	0.34 ± 0.12	6.50 ± 0.32	0.74 ± 0.05	19.26 ± 0.22	7.16 ± 0.08
Fructose	9.37 ± 0.51	0.73 ± 0.02	12.64 ± 0.03	0.93 ± 0.05	17.69 ± 0.62	1.10 ± 0.04	31.58 ± 0.09	13.99 ± 0.21
Acetate	0.57 ± 0.03	1.59 ± 0.11	0.69 ± 0.03	0.70 ± 0.04	0.94 ± 0.41	0.99 ± 0.02	17.86 ± 0.15	12.73 ± 0.17
Lactate	0.47 ± 0.02	10.04 ± 0.19	0.55 ± 0.01	11.30 ± 0.11	0.13 ± 0.03	9.96 ± 0.10	11.70 ± 0.01	21.63 ± 0.07
Ethanol	0.00	12.76 ± 0.27	0.00	12.83 ± 0.30	0.00	11.13 ± 0.06	0.00	7.87 ± 0.01

**Fig. 3:**
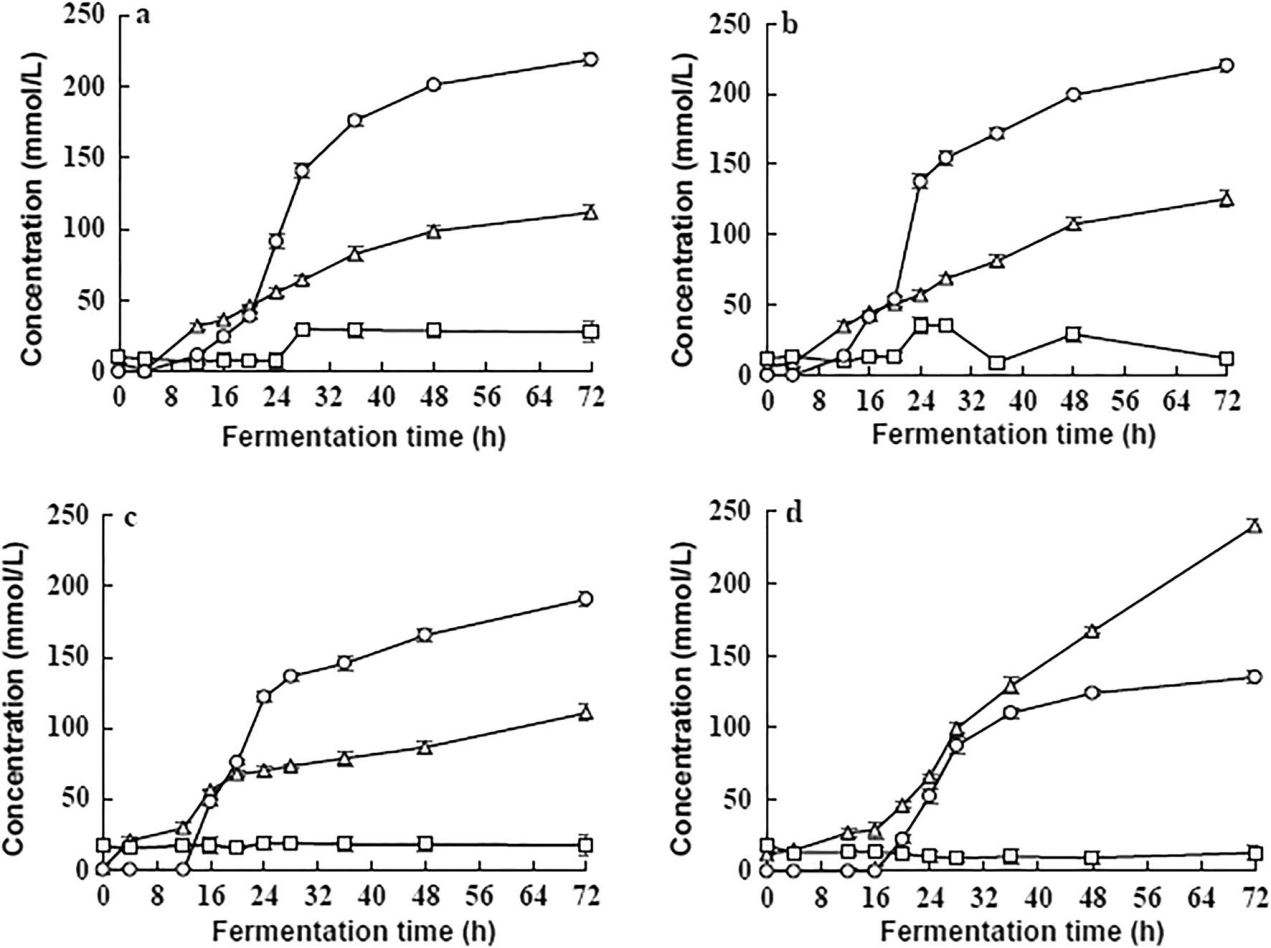
Products in different concentrations of cane molasses. Products tested: Lactate , Acetate , Ethanol . **a** 5 °BX; **b** 7.5 °BX; **c** 10 °BX; **d** 20 °BX

As shown in Fig. 3a, b, the ethanol production of the strain started earlier (4 h) in low concentrated molasses (5 °Bx and 7.5 °Bx) than that at other concentrations (10 °Bx and 20 °Bx). The ethanol yields of strain in 5 °Bx and 7.5 °Bx mediums were similar that they all could reach about 13 g/L after 72 h which were higher (about 2 times) than that in 20 °Bx molasses (7 g/L) (Table 2). The highest ethanol yield was up to 38% (w/w%) in 7.5 °Bx medium (Table 2).

The yield of lactic acid which is another major metabolite was also greatly affected by the concentration. With the increase of molasses concentration, the ratio of ethanol to lactic acid production decreased gradually. In the 5 °Bx molasses, the ratio of ethanol to lactic acid broth was 2.0 (mmol/L: mmol/L) (Table 3), which is the maximum of the four concentrations. In the 20 °Bx molasses, a considerable number of carbohydrates were converted into lactic acid by the strain that the ratio dropped below 1.0 (Table 3, Fig. 3d).

**Table 3:** Carbohydrate utilization ratio and ethanol production ratio in low concentrations of molasses for 72 h

Concentrations	Carbohydrate utilization ratio (w/w%)	Ethanol production ratio (w/w%)	Ethanol: lactate (mmol/L:mmol/L)
5°BX	75.2	35.8	2.0
7.5°BX	56.5	38.0	1.8
10°BX	38.6	35.7	1.7
20°BX	25.6	24.6	0.6

Carbohydrate consumption (g/L) was calculated as Initial carbohydrate content (g/L) minus residual carbohydrate content (g/L). The carbohydrate utilization ratio was calculated using the following equation: Carbohydrate utilization ratio (w/w%) = Carbohydrate consumption (g/L)/Initial carbohydrate content (g/L) × 100%. The ethanol production ratio was calculated using the following equation: Ethanol production ratio (w/w%) = Ethanol yield (g/L)/Carbohydrate consumption (g/L) × 100%

As the carbohydrates utilization rate and the ethanol yield in 5 °Bx molasses were the highest, 5 °Bx is the optimum concentration of molasses for ethanol production.

### Effects of oxygen on the ethanol production

**Fig. 4:**
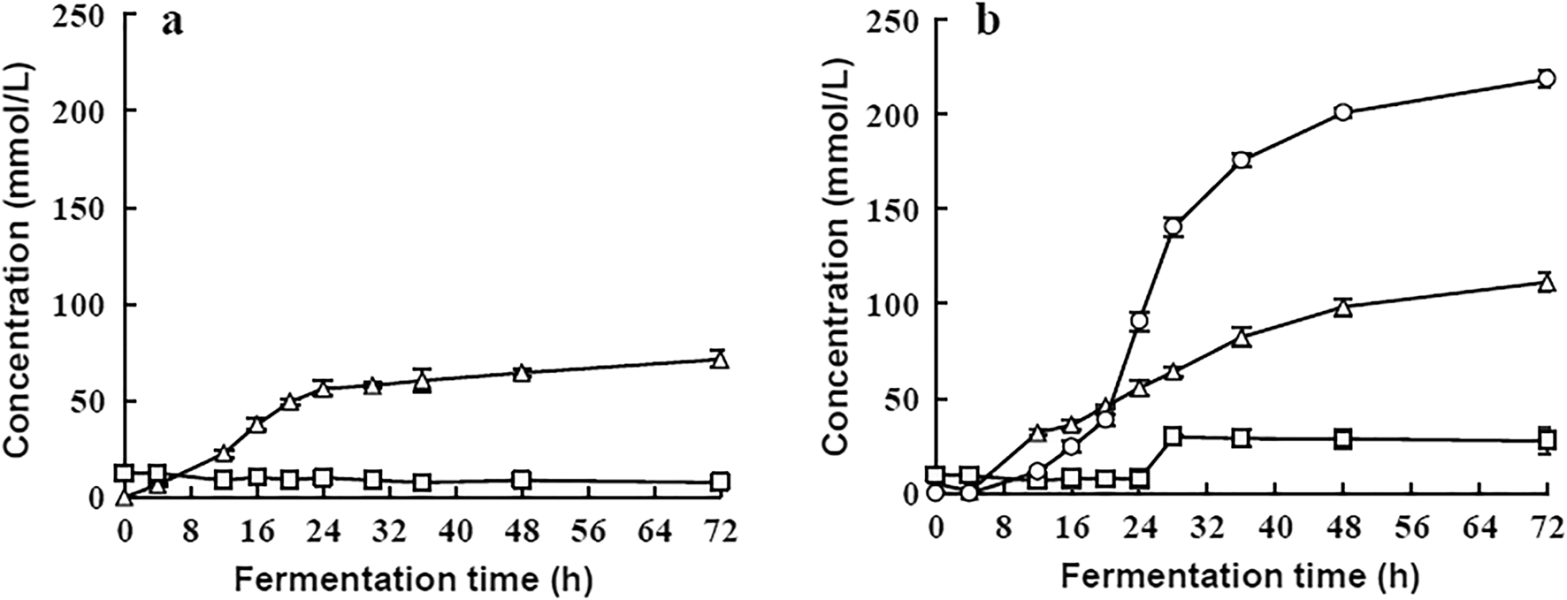
The products in aerobic (**a**) and anaerobic (**b**) fermentation. Products tested: Lactate , Acetate , Ethanol

As shown in Fig. 4, strain E1 could not synthesize ethanol under aerobic conditions; instead, a small amount of lactic acid was produced. Most of the carbohydrates were used to support the growth of cells. In the anaerobic environment, the strain was in a good growth state, and high ethanol content was obtained in the end product of carbon source metabolism.

### Effects of controlled and uncontrolled pH cultures on the growth and metabolism

**Fig 5:**
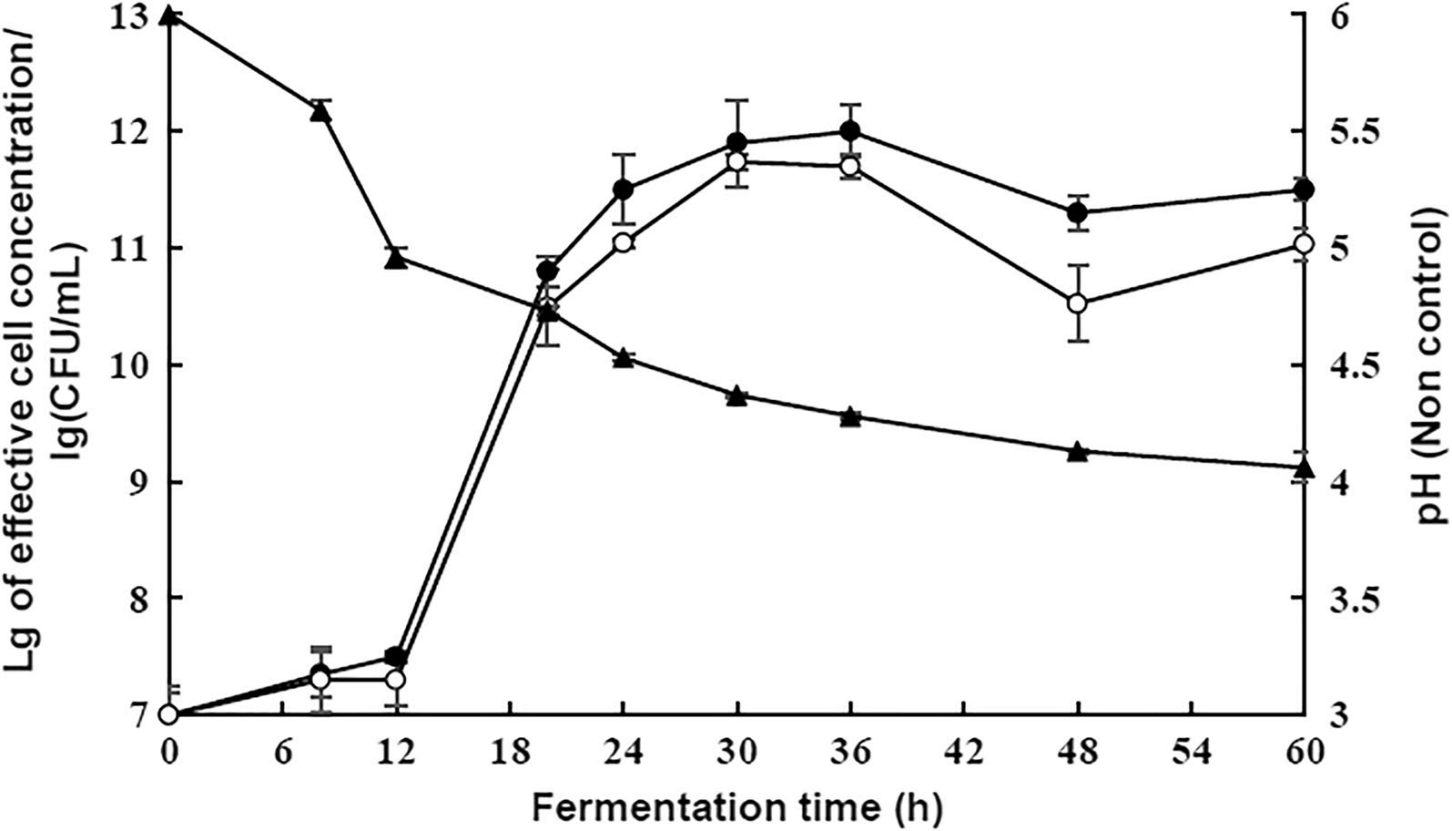
Effect of pH control  and non-pH control  on the growth of strain. pH changes  under non-pH control

In the unregulated pH state, the pH dropped rapidly (from 6.0 to 4.5) within 24 h (Fig. 5). Due to the feedback inhibition of lactic acid accumulation on the bacteria’s glucose metabolism, the growth and metabolism of strain were affected. After 36 h of fermentation, the total sugar utilization was only 66.4%, and the sucrose content remained 10.46 g/L after 60 h.

Under the condition of pH control, the growth and metabolism of strain were exuberant mainly in three aspects: firstly, the activity of strain was high (the number of live cells in 36 h was close to 10^12^ CFU/mL); secondly, the glucose metabolism efficiency was high (the strain consumed all the carbohydrates within 60 h), sugar utilization rate was approximately 78.6%, which was higher (12.2%) than that in the non-pH control state at 36 h (Fig. 5, Table 4); and finally, the ethanol yield was about 14.85 g/L at 60 h, which was higher (about 2.70 g/L) than that in the unregulated pH state (Table 4). Therefore, pH control at 6.0 in the fermentation process is suitable for ethanol synthesis of the strain from cane molasses.

**Table 4:** Effect of pH on carbohydrate metabolism

Time (h)	Residual carbohydrate content (g/L)		Ethanol yield (g/L)	
	pH control	non-pH control	pH control	non-pH control
0	60.83±0.79	61.42±0.29	0.00	0.00
36	13.03±0.52	20.63±0.06	13.77±0.24	10.62±0.17
60	0.00	10.46±0.15	14.85±0.08	12.15±0.35

**Fig. 6:**
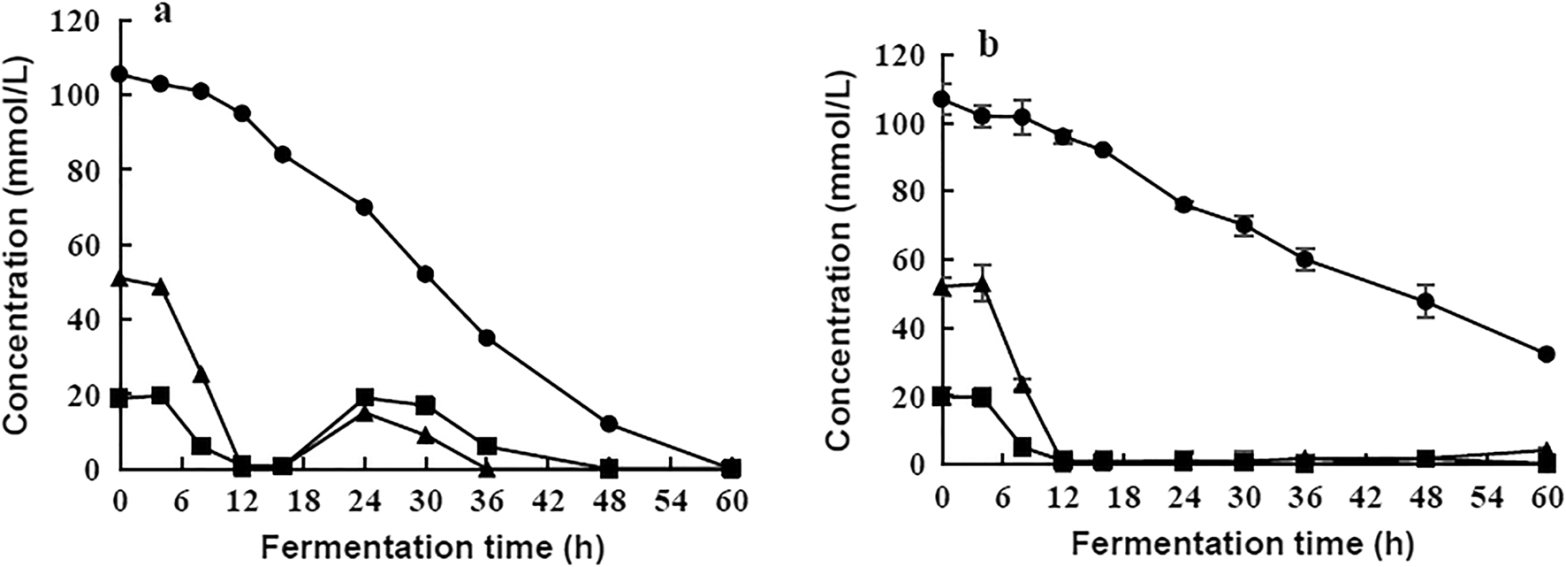
Effect of pH control (**a**) and non-pH control (**b**) on carbohydrate utilization. Carbohydrates tested: Sucrose , Glucose , Fructose

The strain entered the stability growth stage from 24 h and reached the maximum viable amount at 36 h under the condition of regulated pH (Fig. 5). The sucrose metabolism rate of the strain began to decrease from 36 h (Fig. 6a), and ethanol yield was low (only about 1.10 g/L ethanol) during the time frame of 36 h to 60 h (Table 4). Therefore, to reduce costs and increase production efficiency, the optimum fermentation time for ethanol production is 36 h.

### Effects of controlled and uncontrolled pH cultures on the gene expression of key enzymes in carbon source metabolism

Accordingly, the strain consumed glucose and fructose of 5 °Bx molasses within 24 h, and the pH value decreased insignificantly after 24 h (Fig. 5). The expression level of key enzymes in 24 h was determined to evaluate the effect of pH control on carbon metabolism.

**Fig 7:**
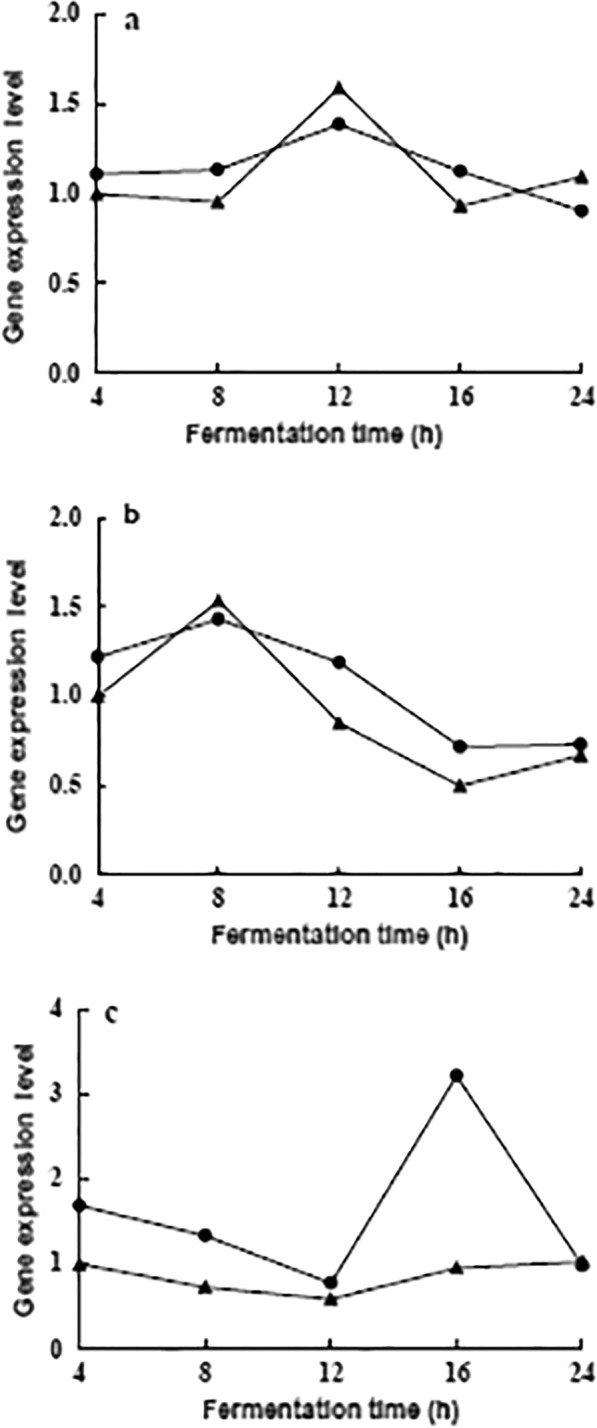
Effect of pH control  and non-pH control  on key enzyme gene expression of carbon source metabolism. **a** GK, glucokinase; **b** INV, invertase; **c** PFK, phosphofructokinase

The relative gene transcription levels of GK, INY, and PFK under pH-regulated and non-pH-regulated mediums were analyzed. The GK gene expression level increased at first and then reduced, and reached the maximum at 12 h (1.25 and 1.59 times of that at 0 h, respectively) under both regulated and unregulated conditions (Fig. 7a). The difference between the two fold lines in Fig. 7a is small, indicating that the effect of the pH change on the GK expression is not significant. The INY and GK genes had similar expression levels, but the maximum value of INY’s appeared at about 8 h (1.17 and 1.54 times of that at 0 h, respectively) under two conditions. Afterward, the INV gene expression level decreased suddenly (only half the initial value at 16 h) under the uncontrolled pH condition (Fig. 7b). In contrast, the PFK gene expression level with the increase of culture time first decreased and then increased. The lowest values were found at 12 h under both conditions. By pH controlled, the expression level of the PFK gene increased significantly (1.90 times of the initial value) at 16 h (Fig. 7c).

**Fig 8:**
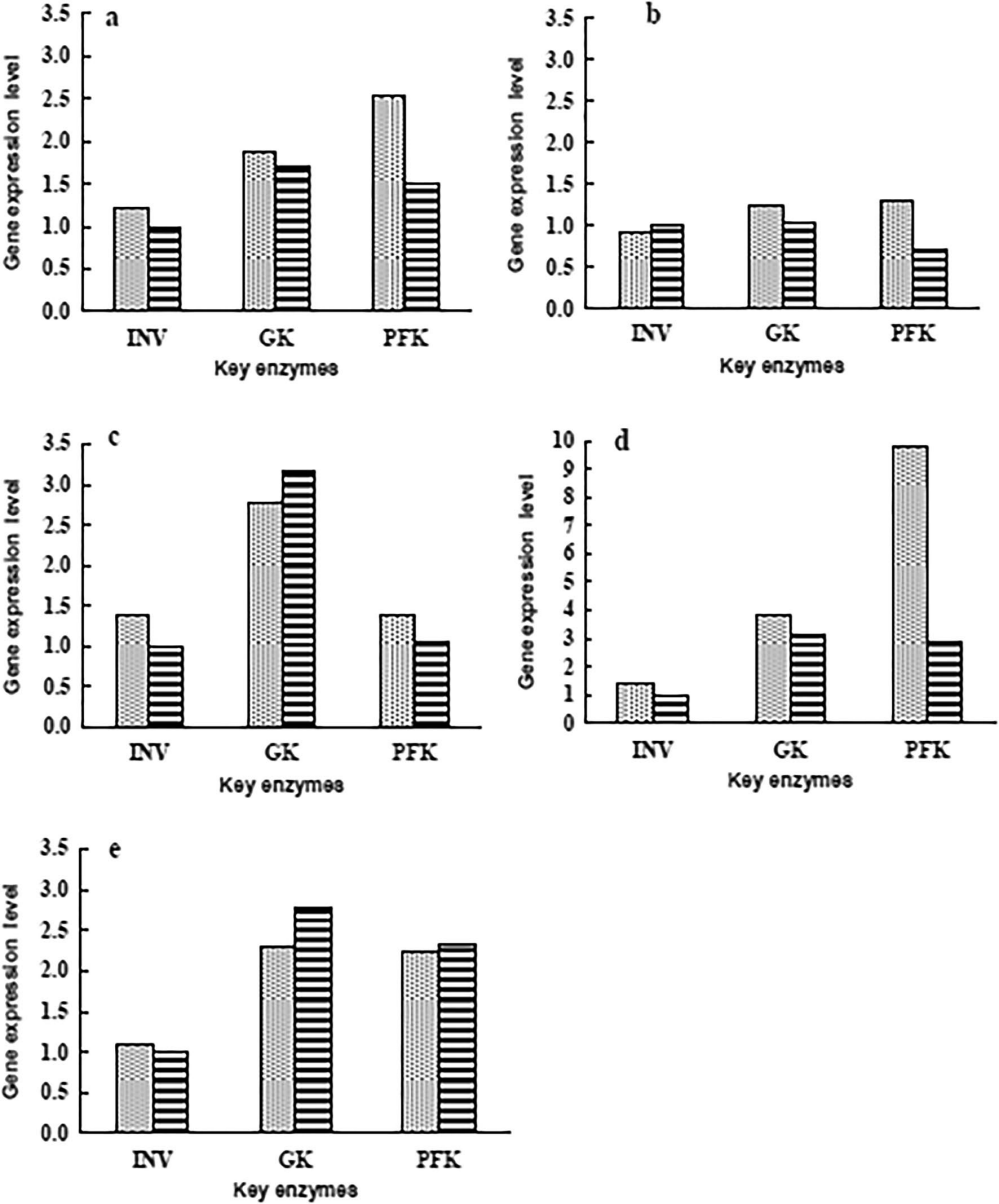
RT-qPCR analysis of key enzyme gene expression under pH control  and non-pH control . INV, invertase; GK, glucokinase; PFK, phosphofructokinase. **a** 4 h; **b** 8 h; **c** 12 h; **d** 16 h; **e** 24 h

Comparing gene transcription levels of three enzymes under two conditions at the same time, except for INV’s gene expression level slightly higher than PFK’s under non-pH control condition at 8 h, the expression levels of INV under both conditions were all lower than those of GK and PFK within 24 h (Fig. 8). The results provide a more detailed theoretical basis, at the molecular level, the strain had the priority metabolic sequence of glucose and fructose, which is consistent with the carbohydrate utilization characteristics of strain (Figs. 2, 6). The expression levels of the PFK gene under controlled pH was significantly higher than those of uncontrolled pH (Fig. 8), indicating the decrease of pH significantly inhibited the PFK gene expression.

## Discussion

### Effects of molasses concentration on the growth and metabolism of the strain

As the main component of sugarcane molasses was sugar (> 50%), molasses’ concentration is directly related to the content of carbohydrates in the medium, the higher its concentration, the higher the osmotic pressure of extracellular environment. The increase in osmotic pressure would lead to the deterioration of the intracellular environment (Bubnová et al. 2014). It can affect the growth and metabolism of the strain, causing the dehydration and inactivation of protein, which hinder cell cycle and inhibit cell growth (Radmaneshfar et al. 2013). Some studies have found that, with the increase of osmotic stress, the cell will actively regulate osmosis that causes intracellular water outflow, shrinking of cells, and even death (Djelal et al. 2017). And microorganisms would increase energy for maintaining the stability of the intracellular microenvironment (enhancing intracellular stress response and tolerance pathway). These will change the metabolic network and regulatory pathways of cells, inhibit the fermentation rate, and change product accumulation (Xu 2010). It was found that the cell growth and pyruvate accumulation rate were inhibited by the increase in osmotic pressure in the pyruvate production (Kamzolova and Morgunov 2016). When the osmotic pressure was increased, the product's yield would significantly decrease (Koganti et al. 2011).

By inference, the metabolization of carbohydrates by strain E1 is in sequence. This characteristic was showed in all the four low concentrations molasses fermentation (Fig. 2), and confirmed by the gene expression results (Fig. 7). Agbogbo et al (2006) used *Pichia pastoris* (*P. stipitis* CBS6054) to ferment a mixed medium of glucose and xylose. It was found that the consumption rate of glucose was higher than that of xylose. This indicated that there is a priority order in carbohydrate metabolism of microorganisms.

### Effects of oxygen on the ethanol production

*Lactiplantibacillus casei* is an aerobic anaerobe like other lactic acid bacteria. Under aerobic conditions, most of the carbohydrates were used for cell proliferation and only a small amount of carbohydrates were converted to lactic acid compared to unsupplemented aerobiosis. Respiration could increase biomass production and alter the central metabolism rerouting pyruvate away from certain metabolic products accumulation (Ianniello et al. 2016). The effect of dissolved oxygen (DO) on the growth of microorganisms is reflected in many aspects, among which the effect on microbial enzymes is an important factor that cannot be neglected. The change of enzyme activity will directly affect the growth and metabolism of the strain, thus affect the types and yields of the products (Zotta et al. 2017; Ricciardi et al. 2019). Ianniello et al (2016) found that the lactate dehydrogenase (LDH) activity was significantly affected by aeration parameters.

Reactive Oxygen Species (ROS), may be produced in the presence of O_2_, can seriously threaten the strain’s survival. Some LAB can be subjected to aerobic growth, with a consequent change in the LAB’s physiological metabolism, including the reduction of biomass and the change of fermentation product types. ROS can destroy cellular proteins, lipids, and nucleic acids, causing cell aging and death (Maresca et al. 2019). Therefore, anaerobic culture is the optimum culture condition for ethanol production by the strain.

### Effects of controlled and uncontrolled pH cultures on the growth and metabolism of the strain

Based on the results of acidity control of fermentation broth, it was found that the change in pH has a significant effect on the growth and metabolism of the strain. This is mainly because fermentation’s organic acid will enter the cytoplasm in the form of diffusion and release proton H^+^ after dissociation, subsequently reducing the intracellular environment’s pH value. The continuous acidification of the intracellular environment destroys DNA structures, the denaturation of proteins, and enzymes’s inactivation. Moreover, changes in pH cause the channel proteins, transporters, and signaling pathway proteins on the membrane to lose their normal function that maintains cell-selective permeability, disturbing the balance of sodium-potassium ions inside and outside cells. Ultimately, the physiological activities being affected results in low production efficiency and poor product quality (Carpenter et al. 2009). In the study of succinic acid production, with the accumulation of succinic acid, the biomass was gradually reduced, and the vitality of the somatic cells was steadily decreased (Andersson et al. 2010). Roa et al (2011) found that the lower the pH value, the lesser the yield of fumaric acid in the fermentation of *Rhizopus oryzae* (Roa Engel et al. 2011).

### Effects of controlled and uncontrolled pH cultures on the gene expression of key enzymes in carbon source metabolism

Many enzymes and multiple metabolic pathways are involved in the regulation of the growth and metabolism of bacterial cells. Under different pH conditions, the strain's ability to metabolize a specific sugar is affected by the activity of key enzymes and the growth of strain. The amount of enzyme gene expression indicates the demand for this enzyme by strain’s metabolism. Therefore, the expression levels of metabolism key enzyme genes can reflect the metabolic level of bacteria to a certain extent.

By in-depth analysis, the three enzymes’ gene expression characteristics under two conditions showed that, on the one hand, the expression levels of GK, INY, and PFK genes with regulated pH were almost more than those under the non-pH control state. The trend is consistent with the change of carbohydrates utilization under two conditions (Fig. 6). This indicates that the increase in acidity inhibits the carbohydrates metabolism of the strain (Virgilio et al. 2017). Furthermore, the genes’ high expression levels under controlled pH demonstrated that the number of sugar molecules transported into the cell is large, which is beneficial to cells’ rapid activation of metabolic function. Therefore, fermentation in the medium with a constant pH of 6.0 is more conducive to the metabolism of the three sugars by the strain; on the other hand, the trend of the gene expression in the two conditions is very similar, indicating that the change of pH does not change the trend of enzyme gene expression.

In conclusion**,** this study has shown the engineered *L. casei* E1 has the abilities to metabolize sucrose, glucose, and fructose of sugarcane molasses into ethanol. In addition, carbohydrates metabolism of *L. casei* E1 can be influenced by cane molasses concentrations, oxygen, and controlled pH. The optimum technological parameters for bioethanol production from molasses by engineering strain: fermenting anaerobically at 37 °C for 36 h incubation in 5 °BX molasses with fermenter's pH controlled at 6.0. The ethanol yield could reach about 13.77 g/L, and carbohydrate utilization percentage is about 78.60%. Some metabolic characteristics of strain have been identified that the genetic strain E1 preferentially ferment glucose and fructose of molasses. And the elevated acidity cannot affect the gene expression trends of three carbohydrate metabolism key enzymes, but inhibit the ability of strain E1 to metabolize fructose.

Overall, this study strengthens the idea that it is feasible to use the engineered *L. casei* strain to produce ethanol. Of course, there is still a long way to go before lactic acid bacteria can be used to produce bioethanol.

## Data Availability

The authors declare that all the data supporting the findings of this study are available within the article.

## References

[CR1] Agbogbo KF, Coward-Kelly G, Torry-Smith M, Wenger KS (2006). Fermentation of glucose/xylose mixtures using Pichia stipitis. Process Biochem.

[CR2] Andersson C, Petrova E, Berglund K, Rova U (2010). Maintaining high anaerobic succinic acid productivity by product removal. Bioproc Biosyst Eng.

[CR3] Baki AS, Bande YM, Bello A (2020). Comparative Studies on Bioethanol Production from Cassava Peels using *Saccromyces Cerevisae* and *Zymomonas Mobilis*. Open Access J Biomed Eng Biosci.

[CR4] Blanco-Míguez A, Fdez-Riverola F, Sánchez B, Lourenço A (2019). Resources and tools for the high-throughput, multi-omic study of intestinal microbiota. Brief Bioinform.

[CR5] Britz TJ, Tracey RP (1990). The combination effect of pH, SO_2_, ethanol and temperature on the growth of *Leuconostoc oenos*. J Bacteriol.

[CR6] Bubnová M, Zemančíková J, Sychrová H (2014). Osmotolerant yeast species differ in basic physiological parameters and in tolerance of non-osmotic stresses. Yeast.

[CR7] Cai H, Rodríguez BT, Zhang W, Broadbent JR, Steele JL (2007). Genotypic and phenotypic characterization of *Lactobacillus casei* strains isolated from different ecological niches suggests frequent recombination and niche specificity. Microbiology.

[CR8] Carpenter CE, Broadbent JR (2009). External concentration of organic acid anions and pH: key independent variables for studying how organic acids inhibit growth of bacteria in mildly acidic foods. Food Sci.

[CR9] Davis CR, Wibowo D, Fleet GH, Lee TH (1988). Properties of wine lactic acid bacteria: their potential enological significance. Am J Enol Viticult.

[CR10] Dittrich CR, Vadali RV, Bennett GN, San KY (2005). Redistribution of metabolic fluxes in the central aerobic metabolic pathway of *E. coli* mutant strains with deletion of the *ackA-pta* and *poxB* pathways for the synthesis of isoamyl acetate. Biotechnol Prog.

[CR11] Djelal H, Chniti S, Jemni M, Weill A, Sayed W, Amrane A (2017). Identification of strain isolated from dates (Phœnix dactylifera L.) for enhancing very high gravity ethanol production. Environ Sci Pollut R.

[CR12] Dziugan P, Balcerek M, Pielech-Przybylska K, Patelski P (2013). Evaluation of the fermentation of high gravity thick sugar beet juice worts for efficient bioethanol production. Biotechnol Biofuels.

[CR13] Edwards CG, Jensen KA (1992). Occurrence and characterization of lactic acid bacteria from Washington State wines: *Pediococcus spp*. Am J Enol Viticult.

[CR14] Fan M, Zhang S, Ye G, Zhang H, Xie J (2018). Integrating sugarcane molasses into sequential cellulosic biofuel production based on SSF process of high solid loading. Biotechnol Biofuels.

[CR15] G-Alegría E, López I, Ruiz JI, Sáenz J, Fernández E, Zarazagaet M, Dizyal M, Torres C, Ruiz-Larrea F (2004). High tolerance of wild *Lactobacillus plantarum* and *Oenococcus oeni* strains to lyophilisation and stress environmental conditions of acid pH and ethanol. FEMS Microbiol Lett.

[CR16] Gold RS, Meagher MM, Hutkins R, Conway T (1992). Ethanol tolerance and carbohydrate metabolism in *Lactobacilli*. J Ind Microbiol Biot.

[CR17] Ianniello RG, Zotta T, Matera A, Genovese F, Parente E, Ricciardi A (2016). Investigation of factors affecting aerobic and respiratory growth in the oxygen-tolerant strain *Lactobacillus casei* N87. PLoS One.

[CR18] Jarboe LR, Grabar TB, Yomano LP, Shanmugan KT, Ingram LO, Olsson L (2007). Development of Ethanologenic Bacteria. Biofuels.

[CR19] Kamzolova SV, Morgunov IG (2016). Biosynthesis of pyruvic acid from glucose by Blastobotrys adeninivorans. Appl Microbiol Biot.

[CR20] Koganti S, Kuo TM, Kurtzman CP, Smith N, Ju L (2011). Production of arabitol from glycerol: strain screening and study of factors affecting production yield. Appl Microbiol Biot.

[CR21] Koryszewska-Bagińska A, Gawor J, Nowak A, Grynberg M, Aleksandrzak-Piekarczyk T (2019). Comparative genomics and functional analysis of a highly adhesive dairy *Lactobacillus paracasei* subsp. *paracasei* IBB3423 strain. Appl Microbiol Biotechnol.

[CR22] Lino FSO, Basso TO, Sommer MOA (2018). A synthetic medium to simulate sugarcane molasses. Biotechnol Biofuels.

[CR23] Liu S, Bischoff KM, Leathers TD, Qureshi N, Rich JO, Hughes SR (2012). Adaptation of lactic acid bacteria to butanol. Biocatal Agric Biotechnol.

[CR24] Livak KJ, Schmittgen TD (2001). Analysis of relative gene expression data using real-time quantitative PCR and the 2^−ΔΔCt^ method. Methods.

[CR25] Maresca D, Filippis FD, Robertiello A, Mauriello G (2019). Metabolic profiling and cold-starvation stress response of oxygen-tolerant Lactobacillus gasseri strains cultured in batch bioreactor. Microorganisms.

[CR26] Matsuoka Y, Kurata H (2017). Modeling and simulation of the redox regulation of the metabolism in *Escherichia coli* at different oxygen concentrations. Biotechnol Biofuels.

[CR27] McAuliffe O, Kilcawley K, Stefanovic E (2019). Symposium review: genomic investigations of flavor formation by dairy microbiota. J Dairy Sci.

[CR28] Meng Q, Yang W, Men M, Bello A, Xu X, Xu B, Deng L, Jiang X, Sheng S, Wu X, Han Y, Zhu H (2019). Microbial Community succession and response to environmental variables during cow manure and corn straw composting. Front Microbiol.

[CR29] Parreiras LS, Breuer RJ, Avanasi NR, Higbee AJ, Reau AL, Tremaine M, Qin L, Willis LB, Bice BD, Bonfert BL, Pinhancos RC, Balloon AJ, Uppugundla N, Sato TK (2014). Engineering and two stage evolution of a lignocellulosic hydrolysate-tolerant *Saccharomyces cerevisiae* strain for anaerobic fermentation of xylose from AFEX pretreated corn stover. PloS One.

[CR30] Qi W, Zhang W, Lu F (2017). Carbon metabolism and transcriptional variation in response to salt stress in the genome shuffled *Candida versatilis* and a wild-type salt tolerant yeast strain. RSC Adv.

[CR31] Radmaneshfar E, Kaloriti D, Gustin CM, Gow NAR, Brown AJP, Grebogi C, Romano MC, Thiel M (2013). From START to FINISH: the influence of osmotic stress on the cell cycle. Plos One.

[CR32] Ricciardi A, Zotta T, Ianniello RG, Boscaino F, Matera A, Parente E (2019). Effect of respiratory growth on the metabolite production and stress robustness of *Lactobacillus casei* N87 cultivated in cheese whey permeate medium. Front Microbiol.

[CR33] Roa Engel CA, van Gulik WM, Marang L, van der Wielen Straathof AJJ (2011). Development of a low pH fermentation strategy for fumaric acid production by *Rhizopusoryzae*. Enzyme Microb Tech.

[CR34] Schwalbach MS, Keating DH, Tremaine M, Marner WD, Zhang Y, Bothfeld W, Higbee A, Grass JA, Cameron Cotten, Reed JL, Sousa LC, Jin M, Balan V, Ellinger J, Dale B, Kiley PJ, Landick R (2012). Complex physiology and compound stress responses during fermentation of alkali-pretreated corn stover hydrolysate by an *Escherichia coli* ethanologen. Appl Environ Microbiol.

[CR35] Suzuki S, Fujita K, Maeno S, Shiwa Y, Kajikawa A (2020). PCR-based screening, isolation, and partial characterization of motile lactobacilli from various animal feces. BMC Microbiol.

[CR36] Takashi K, Sarengaole Hajime T, Bon K (2016). Alcohol-brewing properties of acid- and bile-tolerant yeasts co-cultured with lactic acid bacteria isolated from traditional handmade domestic dairy products from Inner Mongolia. LWT Food Sci Technol.

[CR37] Tanawut N, Poonsuk P, Chonticha L, Supalak S, Pongsak N (2020). Bioconversion of oil palm trunk residues hydrolyzed by rnzymes from newly isolated fungi and use for ethanol and acetic acid production under two-stage and simultaneous fermentation. Waste Biomass Valori.

[CR38] Vinay-Lara E, Wang S, Bai L, Phrommao E, Broadbent JR, Steele JL (2016). *Lactobacillus casei* as a biocatalyst for biofuel production. J Ind Microbiol Biotechnol.

[CR39] Virgilio S, Cupertino FB, Ambrosio DL, Bertolini MC (2017). Regulation of the reserve carbohydrate metabolism by alkaline pH and calcium in Neurospora crassa reveals a possible crossregulation of both signaling pathways. BMC Genomics.

[CR40] Wang TY, Huang CJ, Chen HL, Ho PC, Ke HM, Cho HY, Ruan SK, Hung KY, Wang IL, Cai YW, Sung HM, Li WH, Shih MC (2013). Systematic screening of glycosylation- and trafficking associated gene knockouts in *Saccharomyces cerevisiae* identifies mutants with improved heterologous exocellulase activity and host secretion. BMC Biotechnol.

[CR41] Wang S, LiangG HZ, Jia CF, Zhang BL (2015). Comparative genomics analysis of carbon metabolism of *Lactobacillus casei* 12A. Microbiol China.

[CR42] Welker DL, JoE Hughes, Steele JL, Broadbent JR (2015). High efficiency electrotransformation of *Lactobacillus casei*. FEMS Microbiol Lett.

[CR43] Wu R, Chen D, Cao S, Lu Z, Huang J, Lu Q, Chen Y, Chen X, Guan N, Wei Y, Huang R (2020). Enhanced ethanol production from sugarcane molasses by industrially engineered *Saccharomyces cerevisiae via* replacement of the *PHO4* gene. RSC Adv.

[CR44] Wushke S, Spicer V, Zhang XL, Fristensky B, Krokhin OV, Levin DB, Cicek N, Sparling R (2017). Understanding aerobic/anaerobic metabolism in *Caldibacillus debilis* through a comparison with model organisms. Syst Appl Microbiol.

[CR45] Xin Y, Guo T, Mu Y, Kong J (2018). Coupling the recombineering to Cre-lox system enables simplified large-scale genome deletion in *Lactobacillus casei*. Microb Cell Fact.

[CR46] Xu S (2010) Analysis of the Physiological Mechanisms of Osmotic Stress Tolerance in Torulopsis Glabrata. Doctoral dissertation of Jiangnan University

[CR47] Yao Y, Lv Z, Lin X, Ren M, Zhang B (2011). Application of *Leuconostoc mesenteroide*s subsp. *dextranicum* in the production of cider. China Brew.

[CR48] Zotta T, Parente E, Ricciardi A (2017). Aerobic metabolism in the genus *Lactobacillus*: impact on stress response and potential applications in the food industry. J Appl Microbiol.

[CR49] Zotta T, Ricciardi A, Ianniello RG, Storti LV, Glibota NA, Parente E (2018). Aerobic and respirative growth of heterofermentative lactic acid bacteria: a screening study. Food Microbiol.

